# Functional and genomic characterization of phosphorus-solubilizing enhancement through *Aliirhizobium wenxiniae* SN-4-17

**DOI:** 10.3389/fmicb.2026.1910427

**Published:** 2026-09-09

**Authors:** Chun-Zhi Jin, Naxue Zhang, Ye Zhuo, Taihua Li, Feng-Jie Jin, Jong Min Lee, Long Jin

**Affiliations:** 1 Co-Innovation Center for Sustainable Forestry in Southern China, College of Ecology and Environment, Nanjing Forestry University, Nanjing, China; 2 Department of Biotechnology, Pukyong National University, Busan, Republic of Korea

**Keywords:** *Aliirhizobium wenxiniae*, phosphate-solubilization, pot test, SN-4-17, soybean

## Abstract

**Introduction:**

Phosphorus (P) availability is a major limiting factor in crop productivity due to the predominance of insoluble phosphate complexes in soil.

**Methods:**

A phosphate-solubilizing bacterium, *Aliirhizobium wenxiniae* SN-4-17, was isolated from soybean rhizosphere soil and functionally characterized using physiological, genomic, and biochemical approaches.

**Results:**

Strain SN-4-17 exhibited strong phosphorus-solubilizing activity, releasing up to 652.9 mg/L soluble P at pH 4.0 *in vitro*. Genome analysis (6.41 Mb) revealed genes associated with phosphorus metabolism, including phosphate transport systems, alkaline phosphatases, and CP lyase pathways. Organic acid profiling by HPLC revealed the production of acetic acid, oxalate, and succinic acid as the dominant metabolites, suggesting that organic acid secretion is the primary mechanism responsible for phosphate solubilization. The highest soluble P concentration was observed at pH 4.0 and 30 °C; however, the elevated P solubilization under strongly acidic conditions may have been partly attributable to abiotic dissolution of Ca₃(PO₄)₂. Although no significant effects on plant biomass were observed, the co-application of SN-4-17 with Ca₃(PO₄)₂ in pot experiments resulted in a 106% increase in soil available phosphorus compared to the control.

**Conclusion:**

These findings demonstrate that *A. wenxiniae* SN-4-17 is an efficient phosphate-solubilizing bacterium with potential application to enhance phosphorus availability in agricultural systems.

## Introduction

Soybean (*Glycine max* L.) is a globally important food and agricultural resource (Park et al., 2023). Native to Asia, soybean was domesticated from wild soybean (*Glycine soja* Sieb. & Zucc.) in China approximately 5,000 years ago and has since played a pivotal role in human nutrition (Grassini et al., 2021). Currently, soybean production is primarily directed toward vegetable oil extraction and serves as a key raw material in the food industry, being widely used in products, such as ingredient for vegetable beverages, soy sauce and tofu (Spréa et al., 2024). Along with population growth and socio-economic development, global food demand has increased substantially, posing significant challenges to food security. Many developing regions, including China, have experienced food supply pressure (Liu et al., 2020). China has been the world’s largest soybean importer in recent decades, accounting for nearly 60% of global soybean imports. Approximately 80% of domestic soybean consumption relies on imports to meet the substantial domestic demand (Di et al., 2023). Projections indicate that soybean production in China by will reach 20.73–22.32 million tons and 21.15–27.55 million tons by 2030 under different scenarios, corresponding to self-sufficiency rates of 18.57–24.68%. Despite these projections, China’s soybean industry remains economically uncompetitive globally, primarily due to relatively low productivity and high production costs (Kong et al., 2024). Despite decades of technological advances, soybean yields in China have remained relatively low at approximately 2.0 Mg/ha, representing only about 60% of the yield achieved in the major soybean-producing countries of the Americas (Zheng et al., 2026). Soybean production in China is geographically concentrated in northeastern regions, particularly Heilongjiang and Inner Mongolia Provinces, which represent the major soybean-producing areas (Wang et al., 2020). Even on a global view, soybean production has steadily increased, yield per unit area has remained relatively stagnant over the past decades, indicating the absence of a true “Green Revolution” in soybean breeding (Liu et al., 2020).

Soybean production is governed by multiple interacting factors, including climatic conditions (temperature, photoperiod, and water availability), soil properties (pH and nutrient availability, particularly N, P, K, Mo, Fe, and Zn), biological factors (Rhizobium symbiosis, microbial activity, and pest and disease management), agronomic practices (genotype selection, planting time, plant density, irrigation, and fertilization), and abiotic stress factors such as drought, heat, cold, and flooding (Zimmer et al., 2016; Jabborova et al., 2021; Agegn et al., 2022; Freitas et al., 2022; Wei et al., 2023). Among the various plant growth and development influencing factors, phosphorus is the second most essential macronutrient after nitrogen (Mueller et al., 2019). It typically constitutes approximately 0.2–0.8% of plant dry weight and is a fundamental component of nucleic acids, enzymes, coenzymes, nucleotides, and phospholipids. Although the average total phosphorus content in soil is about 0.05% (w/w), only a very small fraction (approximately 0.1%) is readily available for plant uptake (Bashir et al., 2024).

Plants primarily absorb phosphorus in the form of orthophosphate ions (H₂PO₄^−^ and HPO₄^2−^); however, their concentrations in soil solution are typically in the micromolar range (Johan et al., 2021). In most soils, phosphorus predominantly exists in insoluble forms, such as iron, aluminum, and calcium phosphates, which severely limit its bioavailability (Li et al., 2023). Phosphorus plays a crucial role in a wide range of physiological and biochemical processes, including photosynthesis, root and shoot development, flowering and seed formation, crop maturation, biological nitrogen fixation in legumes, and plant resistance to diseases. Consequently, phosphorus availability is often a major limiting factor for crop productivity (Gerke, 2024). Conventionally, phosphate deficiency in soils is addressed through the application of chemical phosphorus fertilizers (Pang et al., 2024). However, phosphate anions in these fertilizers are highly reactive and are rapidly immobilized through interactions with Ca^2+^, Fe^3+^, and Al^3+^ in the soil, rendering a large proportion of the applied phosphorus unavailable to plants (Li et al., 2024). The formation of insoluble phosphate complexes leads to a low utilization efficiency of chemical phosphorus fertilizers, typically ranging from 5 to 25%. Moreover, long-term application of phosphorus fertilizers can contribute to soil acidification, water pollution, and eutrophication. Therefore, improving the bioavailability of insoluble soil phosphorus has become a critical objective in sustainable agricultural and forestry systems (Timofeeva et al., 2022).

In natural environments, plants interact with a diverse community of root-associated bacteria, commonly referred to as plant growth–promoting rhizobacteria (PGPR), which can establish beneficial relationships that enhance plant growth, yield, and stress tolerance (Wang and Xu, 2026). PGPR enhance plant growth by modulating plant metabolism and improving nutrient acquisition. These effects are achieved through multiple mechanisms, including stimulation of root system development, siderophore production, regulation of phytohormone activity, secretion of bioactive signaling compounds, and phosphate solubilization (Mun et al., 2024; Kolanoś et al., 2026). Members of the genus *Rhizobium* (approximately 170 species), were accommodated to root/stem nodule bacteria, and are widely recognized as symbiotic bacteria of legumes, as PGPR (Young et al., 2023). Rhizobium establishes a mutualistic symbiosis with legumes under nitrogen-limited conditions. Following recognition between plant flavonoids and bacterial nod factors, rhizobia infect root hairs and induce nodule formation (Lepetit and Brouquisse, 2023). These characteristics make them promising biological agents for enhancing plant growth and improving crop yield.

In present research, to identify PGPR and elucidate their functions, a rhizosphere bacterial library was systematically screened. Among these, strain SN-4-17 showed the highest phosphorus-solubilizing activity. And through phylogenetic, physiological, and genomic analysis, the taxonomical position of strain SN-4-17 was indicated to be a member of the genus *Aliirhizobium*. Application of *Aliirhizobium wenxiniae* SN-4-17 significantly enhanced its ability to solubilize inorganic phosphorus via organic acid production rather than phosphatase activity. Collectively, these results suggest that *A. wenxiniae* SN-4-17 is a promising biofertilizer candidate for improving phosphorus availability and enhancing soybean productivity.

## Materials and methods

### Phosphorus-solubilizing bacteria isolation from soybean rhizosphere soil

**Figure 1:**
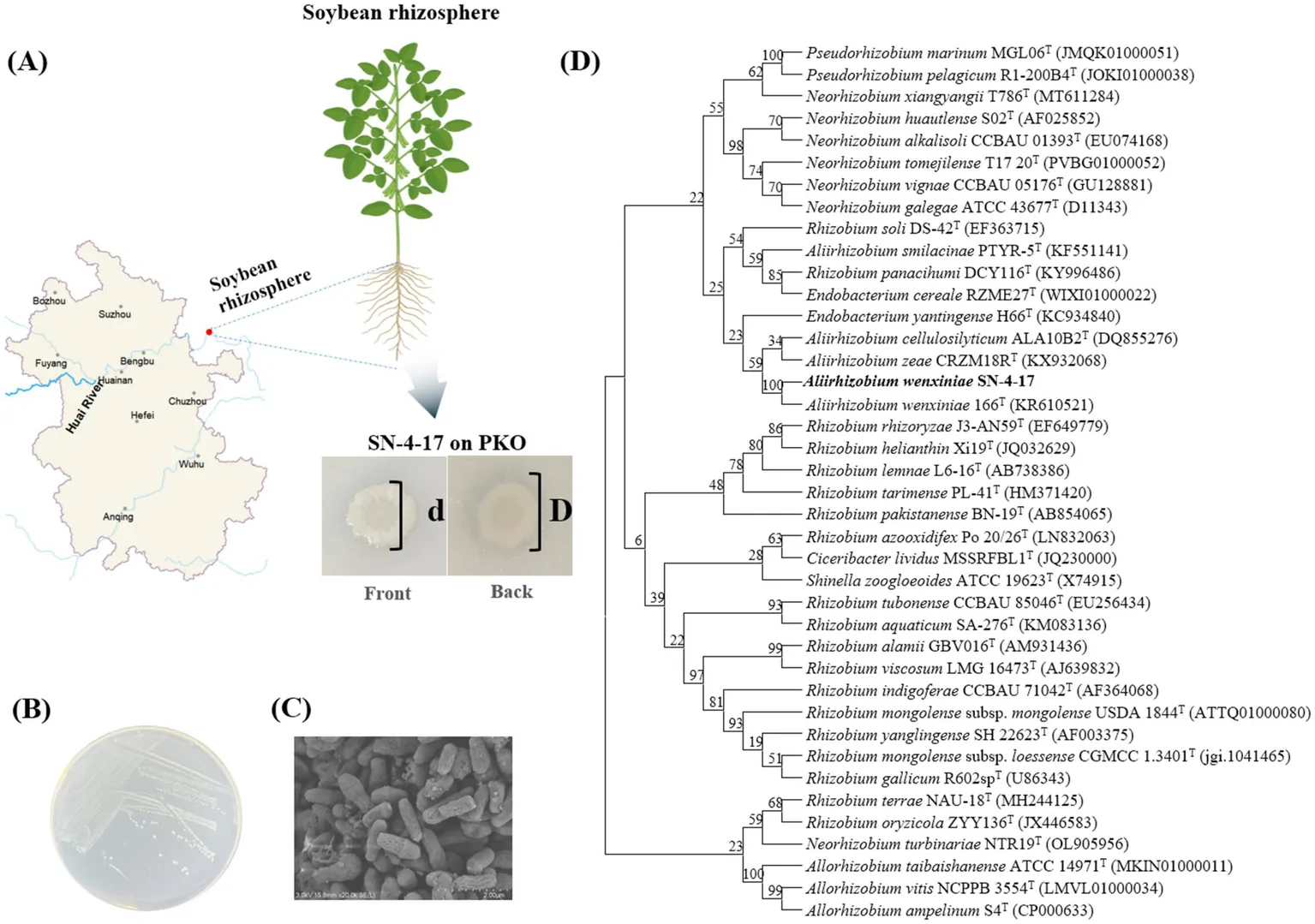
Screening of phosphorus solubilizing microbes from soybean rhizosphere and its characterization. **(A)** Soil sample collection from soybean rhizosphere (map shown on the left) and phosphorus solubilization activity of candidate SN-4-17 on PKO medium (bottom part of **A**). **(B,C)** Colony morphology and scanning electron micrograph of strain SN-4-17 grown on agar plates at 28 °C for 2 days. **(D)** Phylogenetic tree based on 16S rRNA gene sequences showing the taxonomic position of strain SN-4-17 among related taxa. Bootstrap values (%) are indicated at branch nodes.

Soybean rhizosphere soil sample, which was collected from a farmland near Huai River in Anhui, China. Healthy soybean plants were carefully uprooted with their root systems intact under aseptic conditions. Loosely adhering soil was gently removed, and the roots with tightly adhering rhizosphere soil were placed in sterile, sealed bags, labeled, stored at 4 °C to preserve sample integrity, and transported to the laboratory for bacterial isolation. For phosphorus solubilizing bacteria screening, 1 g of soil was serial diluted in distilled 0.9% (w/v) NaCl solution and 100 uL of each dilution was spread onto Pikovskaya (PKO) agar plates (0.3 g NaCl, 0.5 g (NH_4_)_2_SO_4_, 0.3 g MgSO_4_·7H_2_O, 5.0 g CaCO_3_, 0.3 g MnSO_4_·4H_2_O, 0.03 g FeSO_4_·7H_2_O, 10 g Glucose, 0.3 g KCl, 0.2 g Lecithin, 20 g agar, 1 L water, pH 7.2). The base PKO media was added 5.0 g of Ca₃(PO₄)₂ as the phosphorus source. After 2 days incubation at 30 °C, colonies with clear halo zones on PKO medium were screened out as potential phosphorus-solubilizing bacteria. Then the potential candidates were purified through repeated streaking. The isolate designated SN-4-17 was purely isolated from the 10^−7^ dilution PKO plate and routinely cultured at 30 °C on R2A agar (Figure 1A). For long-term preservation and further experimental use, it was stored in a 15% (v/v) glycerol stock solution at −80 °C.

### Genome sequencing, annotation, circular map construction, and ANI analysis

Genomic DNA of strain SN-4-17 was extracted using the FastDNA™ SPIN Kit (MP Biomedicals, Irvine, CA, United States) according to the manufacturer’s instructions. Whole-genome sequencing was performed using a combination of the Oxford Nanopore PromethION 48 platform (Oxford Nanopore Technologies, Oxford, United Kingdom) and the Illumina NovaSeq 6,000 platform (Illumina Inc., San Diego, CA, United States) at Benagen Tech Solutions Co., Ltd. (Wuhan, China). Genome annotation was conducted using the Rapid Annotation using Subsystem Technology (RAST) online server (version 2.0) (Aziz et al., 2008). The complete genome sequence of strain SN-4-17 was visualized using the web-based platform Proksee^1^ (Grant et al., 2023). To assess genomic relatedness, the average nucleotide identity (ANI) values were calculated using Ortho ANI, Original ANI and GGDC algorithms on the Ortho ANI Tool (OAT) provided by EZBio-Cloud server (Lee et al., 2016).

### Phylogenetic analysis

The taxonomic position of strain SN-4-17 was initially determined based on 16S rRNA gene sequence analysis. The 16S rRNA gene was amplified using the universal primer pair 27F (5′-AGAATTTGATCMTGGCTCAG-3′) and 1492R (5′-TACGGYTACCTTGTTACGACTT-3′). The obtained sequence was compared with reference sequences using the EzBioCloud database to determine sequence similarity. 16S rRNA sequences of 39 type strains were retrieved from the blasting results of EzBioCloud. All the 16S rRNA sequences were aligned (gap opening penalty: 15.00, gap extension penalty: 6.66) and trimmed (former and latter part) using MEGA version 11.0 (Tamura et al., 2021). A phylogenetic tree was reconstructed using the neighbor-joining (NJ) algorithm (Saitou, 1987). Evolutionary distances were calculated using the Kimura two-parameter model with 1,000 replication (Jin et al., 2021).

### Physiological, morphological and biochemical analyses of SN-4-17

Cell growth of strain SN-4-17 was evaluated on eight different culture media, including Potato dextrose (PDA), Luria-Bertani (LB), ISP2, ISP4, Bennett, R2A, marine (Difco) and nutrient agar (Liu et al., 2018). Growth characteristics were further examined at different NaCl concentrations (0–10%, w/v), temperatures (4–45 °C), pH values (4.0–12.0, at intervals of 1 pH unit), using R2A as the basal medium for all tests (Zhuo et al., 2023). Colony morphology was observed after 48 h of incubation at 28 °C on R2A agar plates. The cellular morphology subsequently recorded through a scanning electron microscope (Hitachi, Tokyo, Japan). Antibiotic susceptibility was tested using the antibiotic-impregnated containing the following antibiotics (μg per disk): amikacin (30), ampicillin/ sulbactam (20), chloramphenicol (30), erythromycin (30), gentamicin (30), kanamycin (30), lincomycin (15), nalidixic acid (30), rifampicin (30), spectinomycin (25), streptomycin (25), teicoplanin (30), tetracycline (30) and vancomycin (30). Sensitivity was determined by measuring diameter of inhibition zones (Jin et al., 2025). Biochemical characteristics were determined using API ZYM and API 20NE kits (bioMérieux) according to the manufacturer’s instructions. Catalase activity was evaluated by observing oxygen bubble formation after the addition of 3% (v/v) H₂O₂ to bacterial cells (Yang et al., 2020).

### Effect of different culture conditions on phosphorus solubilization

For the evaluation of phosphorus-solubilizing activity, a cell suspension of strain SN-4-17 was prepared and adjusted to an OD₆₀₀ of 1.0. Then 1 mL of the suspension was inoculated into 100 mL of PKO liquid medium. Insoluble phosphates Ca₃(PO₄)₂, AlPO₄, and FePO₄ were individually used as the sole phosphorus sources to evaluate the solubilization capability of the strain. The concentration of soluble phosphorus in the culture supernatant was determined following the molybdenum–antimony colorimetric method (Yu et al., 2022). To investigate the effects of culture conditions on phosphorus solubilization, different carbon sources (glucose, maltose, mannitol, starch, and sucrose), nitrogen sources (ammonium chloride, ammonium sulfate, peptone, potassium nitrate, and sodium nitrate), and initial pH values (4–9) were evaluated. The experiments were conducted independently while all other conditions were kept constant. Cultures were incubated at 30 °C with shaking at 150 rpm for 5 days. Samples were collected every 24 h, centrifuged at 12,000 × g for 10 min at 4 °C, and the resulting supernatants were analyzed for soluble phosphorus concentration and pH. All experiments were performed in triplicate.

### Quantification of organic acids by HPLC

Organic acid production was determined using liquid PKO medium supplemented with the sole phosphorus source Ca₃(PO₄)₂. After cultivation, the cultures were centrifuged at 4 °C, 12,000 × g for 10 min, and the supernatant was filtered through a 0.22 *μ*m nylon filter (Merck Millipore, Darmstadt, Germany). Standard organic acids, including acetic, citric, malic, succinic, fumaric, lactic, and oxalic acids (purity ≥99.0%), were purchased from Sigma-Aldrich (St. Louis, MO, United States). Quantification of organic acids in the filtrate were performed using an UltiMate 3,000 high-performance liquid chromatography (HPLC) system (Thermo Fisher Scientific, Dreieich, Hessen, Germany). The injection volume was 10 *μ*L and the mobile phase was 0.02 mol/L KH₂PO₄ buffer (solvent A, 99%) and methanol (solvent B, 1%), which pH was adjusted to 2.6 using 1 mol/L phosphoric acid. The flow rate was 0.5 mL/min and the chromatographic separation was monitored at 210 nm using a COSMOSIL C18-PAQ column (4.6 × 250 mm; COSMOSIL, Kyoto, Japan) at 30 °C. The HPLC total runtime was continued for 15 min. Eight organic acids standard solutions including acetic, citric, fumaric, lactic, malic, oxalic, succinic and tartaric acids were prepared in 100 mg/L for calibration. Organic acids profiles of SN-4-17 were identified by matching their retention times and quantified through peak areas to the standards. Microbial culture samples were collected at 24, 72, 120, and 168 h. All samples were analyzed in triplicate.

### Plant growth assay

Soil for the pot experiment was obtained from Tianyue Villa, Pei County, Xuzhou City, Jiangsu Province, China. The physicochemical properties of the soil were presented in Table 1. To evaluate the ability of strain SN-4-17 to solubilize insoluble phosphorus in soil, calcium phosphate was supplemented to establish a phosphorus-solubilization assay. Soybean seeds were surface-sterilized with 1% sodium hypochlorite for 5 min, rinsed five times with sterile water, then treated with 75% ethanol for 5 min, followed by an additional five times wash with sterile distilled water. Each treatment consisted of four replicates, with 2 kg of soil per plastic pot. Five seeds were sown per pot in a greenhouse and watered daily using triple-distilled water to maintain adequate moisture. After 7 days, seedlings were thinned to one uniform plant per pot. On day 8, bacterial cultures were grown overnight at 30 °C and 150 rpm, then centrifuged at 4 °C and 5,000 rpm for 15 min. The supernatant was discarded, and the cell pellets were washed twice with sterile distilled water and resuspended to be OD₆₀₀ = 1. The roots were inoculated with 3 mL of the bacterial suspension every 6 days over a 30 days period prior to harvest. The experiment was divided into four groups as shown in Supplementary Table S1. Soil treated with water group was used as control. For the test groups, calcium phosphate, strain SN-4-17, calcium phosphate and strain SN-4-17 together added into the soil. Soil and plant samples were collected to measure soybean plant height, fresh plant biomass, available phosphorus content in the rhizosphere soil, and soil pH after 30 days.

**Table 1:** Physical and chemical properties of the test soil.

pH	Organic carbon (g/Kg)	Organic matter (g/Kg)	Total nitrogen (g/Kg)	Soluble nitrogen (mg/Kg)	Total phosphorus (%)	Available phosphorus (mg/Kg)	Total potassium (g/Kg)	Available potassium (mg/Kg)
8.4	19.3	11.2	1.20	131	0.100	5.4	8.04	154

### Statistical analysis

Plant growth assays were conducted in quadruplicate. The mean ± standard deviation (SD) of the data was presented and each experiment was independently replicated four times. All data were subjected to a two-way analysis of variance (ANOVA) with the software GraphPad Prism 8.0.1. Statistical significance was determined by Tukey Bonferroni multiple range test, and a: *p* < 0.05 and b: *p* < 0.01 was considered to be statistically significant.

## Results

### Construction of a soybean rhizosphere microbial library and screening for PSMs

A total of 95 bacterial strains were isolated from nine soybean rhizosphere soil samples. They were taxonomically classified into several genera, such as *Arthrobacter*, *Microbacterium*, *Paenibacillus*, *Pseudomonas*, *Rhodococcus*, *Rhizobium*, and *Sphingobacterium*, according to the analysis of 16S rRNA sequences. On the basis of the presence of a clear-zone on the PKO agar medium, ten candidates were chosen from the microbial library. Out of the candidates, eight isolates were classified as *Pseudomonas*, while one strain was designated to *Aliirhizobium* and *Paenibacillus*. Figure 1A illustrates that strain SN-4-17 had the second-highest ratio of clear-zone diameter (D) to colony diameter (d), with a value of 1.17. SN-4-17 exhibited stable phosphorus-solubilizing activity among all candidates in liquid PKO medium, where it was capable of dissolving phosphorus to a maximum of 313.98 mg/L.

### Morphological, physiological, phylogenetical, and ANI analysis of SN-4-17

Strain SN-4-17 formed smooth and convex circular with milky white color colony (Figure 1B). Scanning electron microscopy showed rod-shaped cells measuring approximately 1.3 × 0.5 μm (Figure 1C). 1,402 bp 16S rRNA sequences of strain SN-4-17 exhibited the highest similarity to *A. wenxiniae* 166ᵀ (99.5%), *A. zeae* CRZM18Rᵀ (98.4%), followed by *Neorhizobium huautlense* S02ᵀ (98.3%) and *A. terrae* CC-CFT758ᵀ (98.3%). The close phylogenetic relationship between SN-4-17 and *A. wenxiniae* 166ᵀ, *A. zeae* CRZM18Rᵀ was also supported by clustering branch pattern on the neighbor-joining phylogenetic tree (Figure 1D). From genome view, SN-4-17 showed 97.0% Ortho ANI with *A. wenxiniae* DSM 100734 (JACHEG01), while 76.1% with *Neorhizobium huautlense* DSM21817 (PVBM01). And its original ANI and GGDC data were also showing the close relationship with other two strains (Supplementary Figure S1). Strain SN-4-17 could grow on seven different media, including Bennet, PDA, ISP2, NA, LB, MA and R2A (Supplementary Figure S2). Its growth temperature range from 10 to 40 °C (optimal at 28 °C), pH range of 5.0–9.0 (optimal pH 7.0) and 0–2.0% (w/v) NaCl tolerance (optimal 0%) (Supplementary Table S2). Based on API ZYM and 20NE tests, SN-4-17 showed positive enzymatic activity for lipase (C8, C14), cystine arylamidase, *α*-chymotrypasin, *α*-galactosidase, *β*-galactosidase, *β*-glucuronidase, *N*-acetyl-*β*-glucosaminidase, *α*-mannosidase and *α*-fucosidase (Supplementary Table S3). The strain could assimilate D-Glucose, L-arabinose, D-mannose, D-mannitol, N-acetyl-glucosamine, maltose, potassium gluconate and malate. It was positive for β-glucosidase and β-galactosidase (Supplementary Table S4).

### Prediction of secondary metabolites and phosphate metabolism involved genes

**Figure 2:**
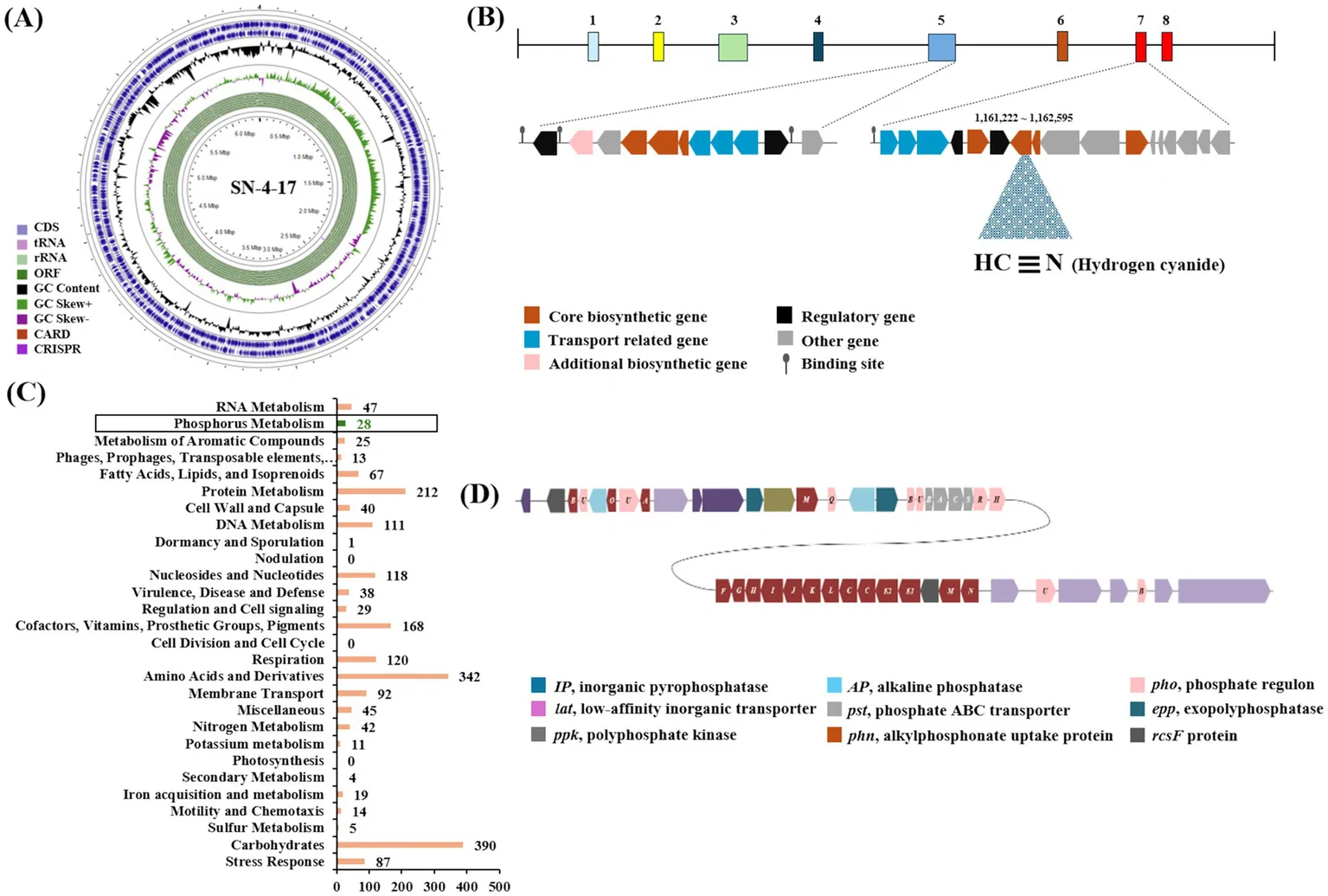
**(A)** Genome circular map. **(B)** Secondary metabolites prediction through antiSMASH analysis. **(C)** RAST-based gene annotation of the *A. wenxiniae* SN-4-17 primary metabolic pathways and stress/defense functions, highlighting its metabolic versatility and biosynthetic potential. **(D)** Phosphorus solubilization related genes annotation from SN-4-17 genome.

The circular genome map of SN-4-17, including coding sequences (CDSs), tRNA, rRNA, open reading frames (ORFs), GC content, GC skew, CARD annotations and CRISPR/Cas clusters were presented in Figure 2A. The genome is 6,409,551 bp, comprising 16 rRNAs (6 5S, 5 16S, 5 23S rRNAs), 63 tRNAs, and an N50 value of 3,973,541 bp (Supplementary Table S5). The antiSMASH analysis of the strain SN-4-17 genome identified eight secondary metabolite biosynthetic gene clusters. Two potentially hydrogen cyanide (HCN) biosynthesis gene clusters were observed (Figure 2B).

RAST based functional annotation of the *A. wenxiniae* SN-4-17 genome showing the highest numbers of genes assigned to carbohydrate (390), amino acid and derivatives (342), protein metabolism (212) and cofactor, vitamins, prosthetic group, pigments (168). Totally 92 genes were assigned to the membrane transport and 28 genes were predicted relate to phosphorus metabolism (Figure 2C). Expansion these phosphorus assimilation and solubilization related genes, they were inorganic pyrophosphatase (*IP*), alkaline phosphatase (*AP*), exopolyphosphatase (*ep*), phosphate-responsive regulators (*pho*), low-affinity inorganic transporter (*lat*), phosphate ABC transporter (*pst*), alkylphosphonate uptake protein (*phn*), and polyphosphate kinase (*ppk*). The genome contains numerous alkaline phosphatases (*AP*) that facilitate the hydrolysis of phospholipids and the release of inorganic phosphorus. Additionally, genes that regulate the phosphorus deprivation response (*phoBRU*) were identified. The genome of SN-4-17 contains all the predicted C-P lyase genes that are required for phosphonate degradation, as well as the additional putative auxiliary functions. These genes consist of a transcriptional regulator (*phnF*), phosphonate ABC transporters (*phnCE1E2*), a metal-dependent phosphonate hydrolase (*phnM*), a phosphonates transport ATP-binding protein (*phnKLN*), and triphosphate phosphonylase components (*phnGHIJ*). These genes, together with those responsible for phosphorus absorption (*phnAB*) and transport (*pstABCS*), control alkaline phosphatase gene expression, especially under low-phosphorus environments. These genes may promote plant growth and bacterial phosphorus solubilization were predicted (Figure 2D).

### Phosphatase activity determination and factors affecting phosphorus solubilization

**Figure 3:**
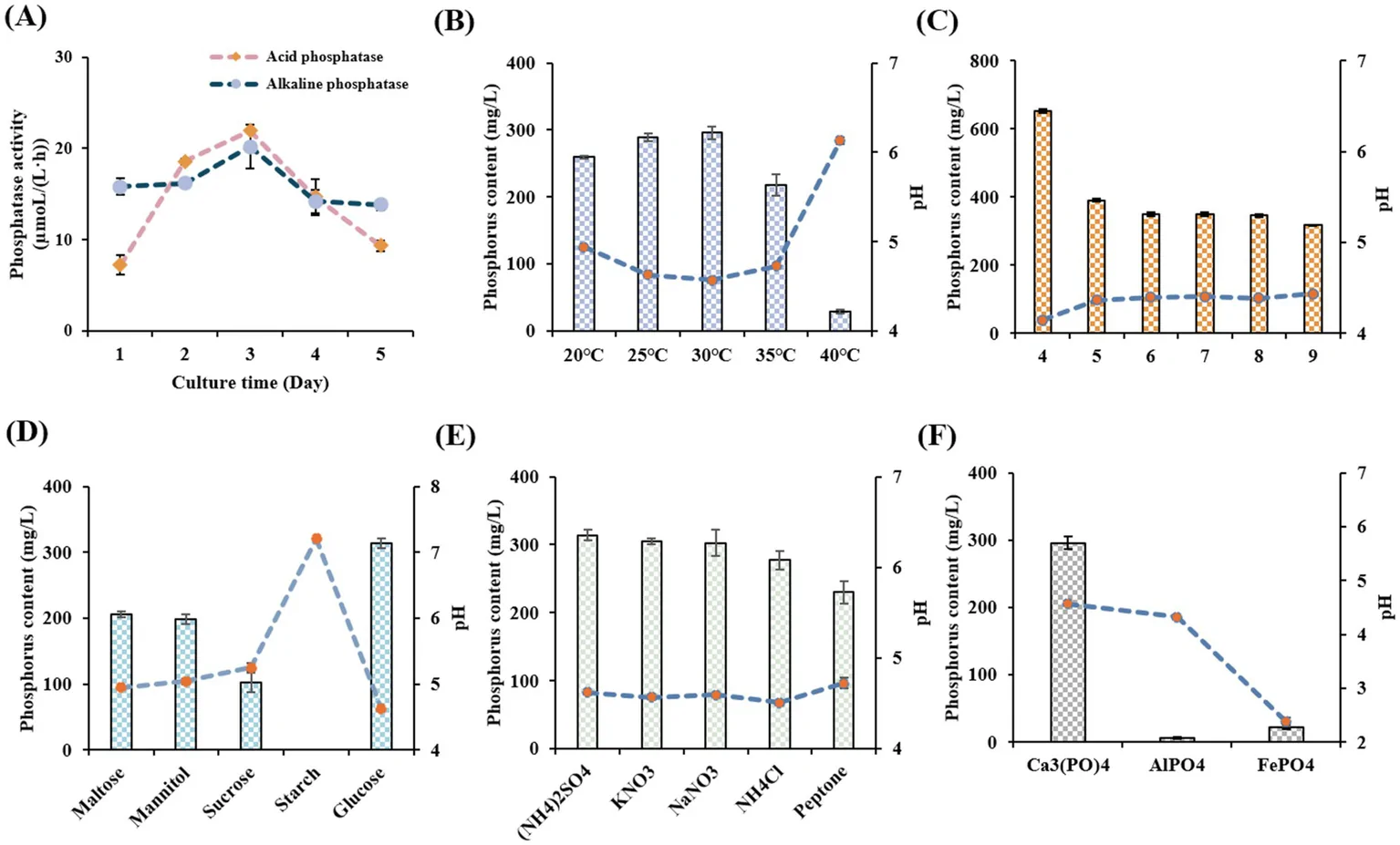
**(A)** Acid and alkaline phosphatase activity during 5 days of cultivation. **(B–F)** Effects of environmental and nutritional factors on phosphorus solubilization by strain SN-4-17, including **(B)** temperature, **(C)** initial pH, **(D)** carbon sources, **(E)** nitrogen sources, and **(F)** phosphorus sources. Data are presented as mean ± standard deviation.

Environmental factors such as culture duration and temperature, types of carbon, nitrogen, phosphorus sources and pH of media could affect microbial phosphorus-solubilizing activity (Li et al., 2022). The effects of different culture conditions on phosphorus solubilization by strain SN-4-17 were investigated (Figure 3). Cultivation in PKO liquid medium for 5 days, SN-4-17 exhibited different enzyme activity patterns of acid and alkaline phosphatase. As shown in Figure 3A, both acid phosphatase and alkaline phosphatase activities increased during the early stage of cultivation and reached their maximum values on day 3, followed by a gradual decline thereafter. Acid phosphatase activity peaked at approximately 21.9 *μ*mol/(L·h), whereas alkaline phosphatase activity reached about 20.2 *μ*mol/(L·h). Phosphatase activities were calculated based on the standard curve shown on Supplementary Figure S3.

### Temperature and pH effects on phosphorus solubilization

Phosphatases are widely produced by microorganisms, animals, and plants, and typically exhibit optimal activity at temperatures of 45–60 °C and pH values between 4.5 and 6.0 (García-Berumen et al., 2025). SN-4-17 showed phosphorus-solubilizing activity across a wider temperature range of 20–40 °C. The highest phosphorus concentration was observed at 30 °C (approximately 295 mg /L), whereas both lower and higher temperatures reduced phosphorus release, especially while the temperature was up to 40 °C, the phosphorus content decreased sharply (Figure 3B). Phosphorus solubilization was strongly influenced by the initial pH of the medium. The highest phosphorus concentration was detected under highly acidic conditions (pH 4.0), reaching approximately 652.9 mg/L. As the initial pH increased toward alkaline conditions, phosphorus solubilization decreased markedly, declining to 391.1 mg/L at pH 5.0, and then tend to be stably decreased (Figure 3C). These results indicate that acidic conditions favor the phosphorus-solubilizing ability of the strain, whereas neutral to alkaline conditions suppress phosphorus release.

### Carbon, nitrogen and phosphorus sources effects on phosphatase activity

Carbon and nitrogen sources could influence microbial growth, metabolism and consequently affect enzyme activity. Therefore, five carbon sources and five nitrogen sources were evaluated to assess their effects on phosphorus solubilization. Different carbon sources showed distinct effects on phosphorus solubilization. Among the tested carbon sources, glucose resulted in the highest phosphorus concentration (approximately 314.0 mg/L), whereas sucrose produced relatively lower level and starch could not solubilize phosphorus (Figure 3D). Nitrogen sources also significantly influenced phosphorus solubilization. The highest phosphorus concentration was obtained with (NH₄)₂SO₄ (approximately 314.0 mg/L), followed by KNO₃ and NaNO₃, while peptone showed the lowest phosphorus-solubilizing efficiency (approximately 230.1 mg/L) (Figure 3E). The type of insoluble phosphorus source further affected solubilization efficiency, because it is the substrate of the phosphatse. Among the tested sources, Ca₃(PO₄)₂ showed the highest phosphorus release (approximately 296 mg/L), whereas AlPO₄ and FePO₄ resulted in substantially lower soluble phosphorus levels (Figure 3F).

### Organic acids productivity of SN-4-17

Organic acid production represents a key mechanism underlying bacterial solubilization of insoluble phosphorus. The secretion of organic acids reduces the pH of the surrounding environment, thereby promoting the dissolution of inorganic phosphate minerals. Furthermore, these acids are capable of chelating metal ions, including Ca^2+^, Mg^2+^, Fe^3+^, and Al^3+^, which further enhances phosphorus availability (Xie et al., 2021). Organic acids production by strain SN-4-17 during 7 days cultivation period in liquid PKO medium was profiled using High-Performance Liquid Chromatography (HPLC).

**Figure 4:**
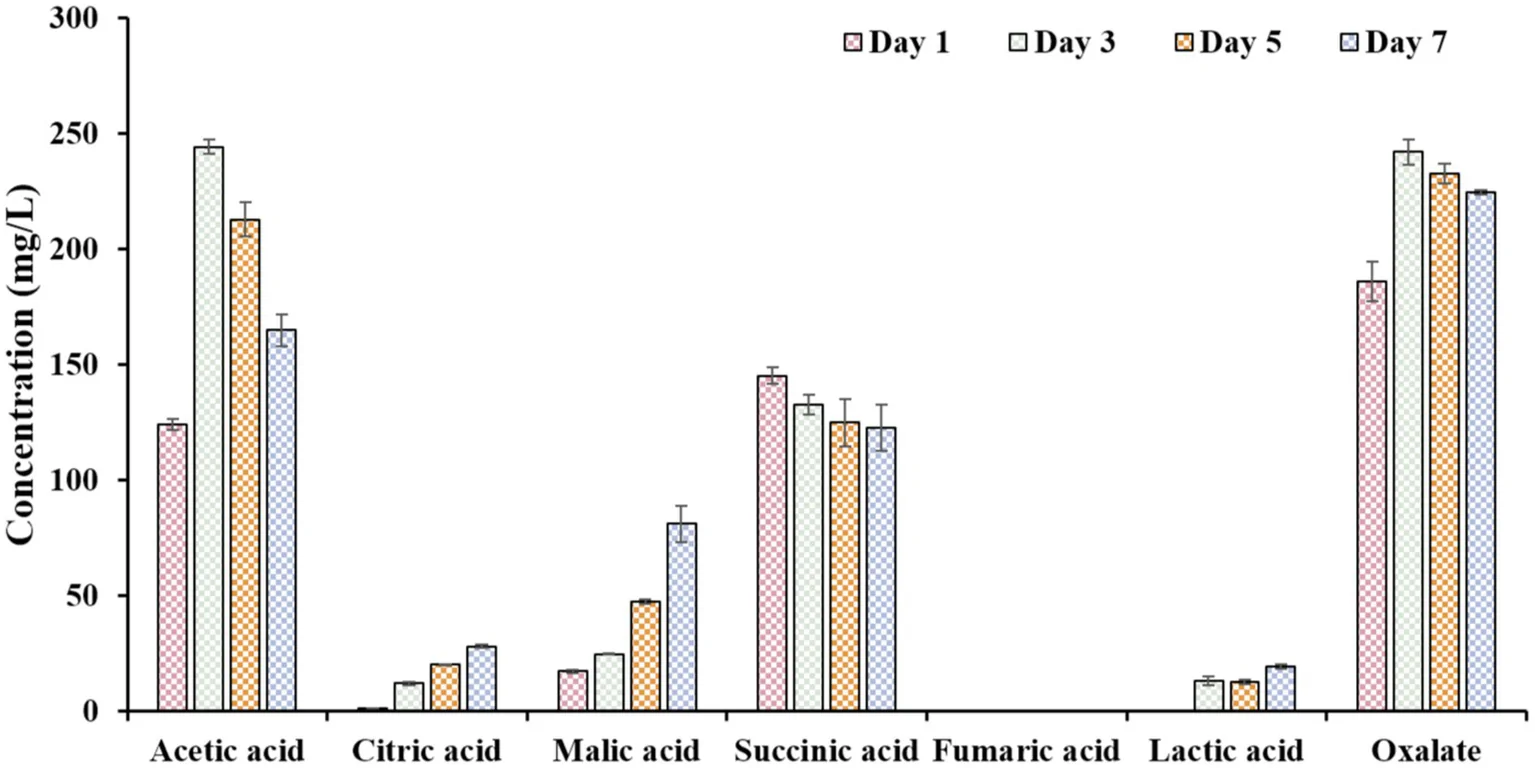
Quantification of organic acids produced by strain SN-4-17 over 7 days of cultivation, including acetic, citric, malic, succinic, fumaric, lactic, and oxalic acids. Data are presented as mean ± standard deviation.

A range of organic acids, including acetic acid, citric acid, malic acid, succinic acid, lactic acid, and oxalate, were detected, whereas fumaric acid was negligible throughout the incubation period. Among these metabolites, acetic acid and oxalate were the predominant organic acids. Acetic acid increased from 124.1 mg/L on day 1 to a maximum of 244.1 mg/L on day 3, followed by a gradual decline to 164.8 mg/L on day 7. Similarly, oxalate reached its highest concentration on day 3 (241.8 mg/L) and remained at relatively high levels thereafter. Succinic acid was produced at comparatively high concentrations at the early stage (145.0 mg/L on day 1) but decreased gradually over time. In contrast, malic acid and citric acid exhibited a continuous increase during cultivation, reaching 81.2 and 28.1 mg/L, respectively, by day 7. Lactic acid was detected at relatively low concentrations (Figure 4). Overall, these results demonstrate that strain SN-4-17 produces diverse organic acids, with acetic acid and oxalate as the dominant metabolites during growth.

### Effects of *A. wenxiniae* SN-4-17 on soybean by pot test

Many reports about microbes (Firmicutes, Actinomycetes, Proteobacteria) could solubilize insoluble phosphorus through different mechanisms such as organic acid production, phosphatase secretion, siderophore release, and so on (Sun et al., 2026). But whether these kinds of activities could be still effective under heterogeneous and harsh natural soil or farm environments is challenging. Lots of microbes exhibited high efficiency under controlled laboratory conditions, failed to demonstrate comparable performance in natural soil environments. To evaluate the practical applicability of *A. wenxiniae* SN-4-17, its capacity to solubilize calcium phosphate and promote soybean growth was investigated through a pot experiment.

**Figure 5:**
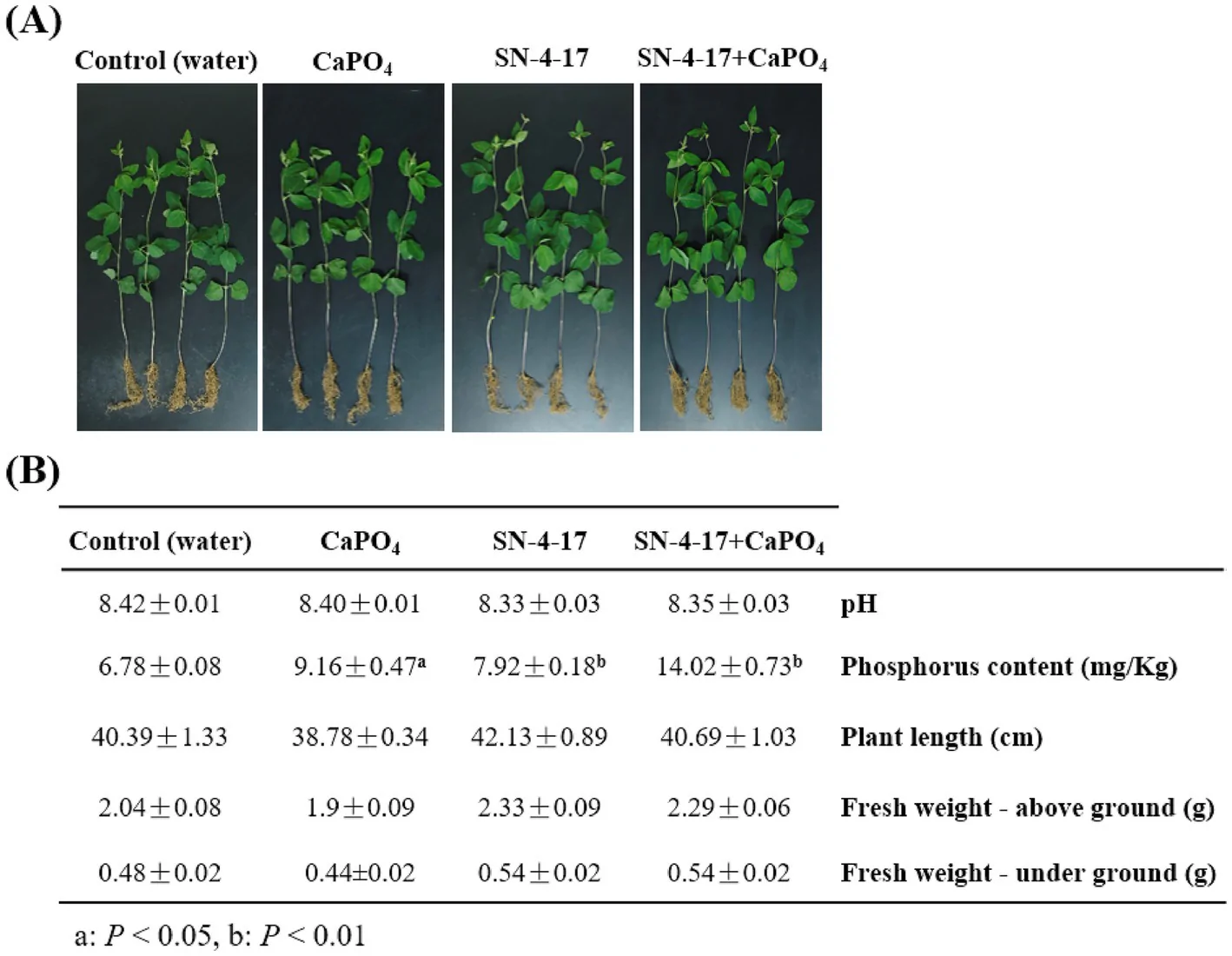
The effects of SN-4-17 treatment on soybean in pot tests. **(A)** Picture of soybean from water, CaPO_4_, SN-4-17, SN-4-17 and CaPO_4_ co-treatment group. **(B)** Phosphorus content, pH, plant length and fresh weight (underground, aboveground) changes in four different groups. a: *p* < 0.05, b: *p* < 0.01.

Soybean plants growth among treatments was shown in Figure 5A. Soil pH remained stable across treatments, ranging from 8.33 ± 0.03 to 8.40 ± 0.01, with no significant changes. Phosphorus content was significantly increased from 6.78 ± 0.08 mg/Kg to 9.16 ± 0.47 mg/Kg and 7.92 ± 0.18 mg/Kg following CaPO₄ and SN-4-17 treatments, respectively. The combined treatment could enhance phosphorus solubilization to be 14.02 ± 0.73 mg/Kg, significantly higher than single treatment. Three treatment groups of plant length ranged from 38.78 ± 0.34 to 42.13 ± 0.89 cm, above-ground fresh weight was 1.9 ± 0.09 to 2.29 ± 0.06 g, under-ground fresh weight was 0.44 ± 0.02 to 0.54 ± 0.02 g which has no significant change comparing with control group. The length and fresh weight of plants (above-ground and under-ground) were not substantially impacted by phosphorus increases during these periods (Figure 5B).

## Discussions

In rhizosphere soil, an abundant of PGPR are present, especially phosphate-solubilizing microorganisms (PSMs), which play a key role in enhancing plant growth and improving crop productivity (Wang et al., 2017). PSMs mobilize phosphorus primarily through organic acid production, proton (H^+^) excretion, and phosphatase-mediated mineralization of organic phosphorus (De Zutter et al., 2022).

*In vitro* screening of PSMs frequently involves quantitative validation, which is followed by medium pH changes. However, pH-based methodologies may generate false positives by selecting strains that do not phosphate-solubilize. Similarly, the formation of a halo zone on agar that contains insoluble phosphorus is not a reliable indicator. In addition, these methods may preclude the use of alternative solubilization mechanisms, such as hydrogen cyanide production, by bacteria (Quijia-Pillajo et al., 2026). Microbial libraries were established in this investigation by isolating samples from soybean rhizosphere soil. The objective of this research is to screen PSMs from the microbial library through checking organic acid production, phosphorus solubilization on PKO agar media and combining genome analysis to address the phosphorus solubilization mechanisms. In spite of the fact that the genome prediction through antiSMASH identified the essential genes for hydrogen cyanide biosynthesis. The gene cluster, located at positions 1,161,222 to 1,162,595 bp, exhibited a typical gene organization consisting of core biosynthesis, regulatory elements, transport-related, and additional associated genes. Binding sites were also identified within the cluster, indicating potential transcriptional regulation. The presence of multiple gene categories suggests the potential of the production of HCN production in strain SN-4-17 (Figure 2B). The further work is needed to confirm the existence of hydrogen cyanide in the culture broth of SN-4-17.

Many *Rhizobium* species that are associated with plants have been reported to be capable of producing ACC deaminase, IAA, phosphorus solubilization, exopolysaccharide, and siderophore, thereby enabling them to function as PGPR (Kamran et al., 2017; Alinia et al., 2022; Maslennikova et al., 2022; Cruz et al., 2023; Xie et al., 2026). In this way, beneficial microbes convert insoluble phosphorus into forms readily available for plant uptake (Ma et al., 2025). The use of PSMs offers a sustainable and eco-friendly alternative to chemical fertilizers, representing a promising approach to enhance soil phosphorus availability and improve crop productivity (Sarmah and Sarma, 2023). However, antiSMASH analysis of the SN-4-17 genome did not identify any biosynthetic gene clusters potentially involved in the production of metabolites associated with these plant growth-promoting traits.

Phosphatase activity of *A. wenxiniae* SN-4-17 was detected on Figure 1A, but the activity was generally very low, around 10–20 umoL/(L·h), so it may not be the main mechanism of phosphorus solubilization (Figure 3A). The secretion of low-molecular-weight organic acids represents another predominant biochemical strategy employed by PSMs. These acids—including oxalic, citric, gluconic, succinic, α-ketoglutaric, fumaric, malic, lactic, and acetic acids—acidify the surrounding soil environment and facilitate the conversion of insoluble phosphorus compounds into bioavailable forms (Wei et al., 2018). Among these, acetic acid has been reported to exert a particularly strong effect, increasing available phosphorus by up to 185.77% (Zhao et al., 2024), Oxalic acid also demonstrates high efficiency; for example, it enhanced phosphorus release from sewage sludge biochar (SSB300 and SSB500) by 103- and 600-fold, respectively, compared to water extraction controls (Santos et al., 2024). In addition, microbial production of succinic acid (~10.97 g/L) has been shown to contribute significantly to phosphorus solubilization, with P release reaching approximately 91–103 mg/L from Ca₃(PO₄)₂ (Zhang et al., 2023).

The production of organic acids, such as acetic acid, succinic acid, and oxalate, by SN-4-17 was approximately 150 mg/L during the 7 days cultivation period. Acetic acid and oxalate exhibited a rise-and-fall pattern during the seven-day production of organic acids, while succinic acid is progressively decreasing. Therefore, the effects of various acids are contingent upon the specific maturation stage of the cell. The acid production was consistently accompanied by pH values below 5, indicating that the soil could potentially become acidified. Additionally, these organic acids act as effective chelating agents for divalent cations such as aluminum (Al), iron (Fe), and calcium (Ca), thereby facilitating the release of soluble phosphate from otherwise insoluble mineral complexes (Hou et al., 2018; Bargaz et al., 2021). In most soils, phosphorus predominantly exists in insoluble forms complexed with Ca, Al, or Fe, which significantly limits its bioavailability for plant uptake (Oteino et al., 2015). Soil pH plays a critical role in regulating the speciation and availability of inorganic phosphorus. Under acidic conditions, phosphorus is primarily present as insoluble iron and aluminum phosphates or associated with mineral matrices. In contrast, alkaline soils are typically characterized by the predominance of calcium phosphate complexes, which also exhibit low solubility (Zuluaga et al., 2023). The phosphatase from strain SN-4-17 was just only could solubilize Ca_3_(PO_4_)_2_, not AlPO_4_ and FePO_4_ (Figure 3F). Moreover, its phosphorus-solubilizing activity was highest at pH 4.0. These findings suggest that SN-4-17 is primarily adapted to solubilizing calcium-bound phosphate under acidic conditions, which may limit its effectiveness in soils where phosphorus is predominantly present as iron or aluminum phosphates. But on the other point, the pH 4.0 is also a factor which could affect phosphorus solubilization.

Field and pot experiments have demonstrated that application of PSMs could enhance phosphorus solubilization by up to 50% to maintain crop production (Xing and Wang, 2024). In the present study, co-application of SN-4-17 with Ca₃(PO₄)₂ increased soil soluble phosphorus by 106.8% compared with the water-treated control (Figure 5B). However, this increase in phosphorus availability did not result in significant changes in soil pH, plant length, or fresh biomass (both above- and belowground). *In vitro* assays suggested that phosphorus solubilization by SN-4-17 was primarily associated with organic acid secretion rather than phosphatase activity. In contrast, no significant change in soil pH was observed in the pot experiment, indicating that the underlying mechanism in soil may differ from that observed under laboratory conditions. These results suggest that further optimization of microbial application and calcium phosphate supplementation is required to enhance *in situ* efficacy. Additional experiments evaluating dry biomass, phosphorus uptake, root development, and long-term performance are needed to further validate the effects of strain SN-4-17.

In conclusion, this study identified *A. wenxiniae* SN-4-17 as an efficient phosphate-solubilizing bacterium capable of mobilizing insoluble calcium phosphate through organic acid production. Although enhanced phosphorus availability was observed in pot experiments, no significant improvement in soybean growth was detected. These results indicate that increased phosphorus solubilization does not necessarily translate into enhanced plant performance, highlighting the complexity of plant-microbe-soil interactions. Future studies should investigate colonization efficiency, interactions with indigenous soil microbiota, nutrient status, and additional plant growth-promoting mechanisms to better assess the strain’s agricultural value.

## Data Availability

The whole genome sequence of strain SN-4-17 was deposited in GenBank/EMBL/DDBJ under the accession number PRJNA1438419. The strain was deposited in the China General Microbiological Culture Collection Centre with the number of CGMCC1.62083.

## Footnotes

1
https://proksee.ca
