# Supplementary material for: Functional and genomic characterization of phosphorus-solubilizing enhancement through Aliirhizobium wenxiniae SN-4-17
Source: Front Microbiol. 2026 Sep 9;17:1910427. doi: 10.3389/fmicb.2026.1910427 (PMC13597829; doi:10.3389/fmicb.2026.1910427)
Supplement: Supplementary file 1 [file Data_Sheet_1.pdf]

SUPPORTING MATERIALS

Functional and genomic characterization of phosphorus-solubilizing enhancement through *Aliirhizobium wenxiniae* SN-4-17

Chun-Zhi Jin[1, 2], Naxue Zhang[1], Ye Zhuo[1], Taihua Li[1], Feng-Jie Jin[1], Jong Min Lee[2]*, Long Jin[1]*

[1] Co-Innovation Center for Sustainable Forestry in Southern China, College of Ecology and Environment, Nanjing Forestry University, Nanjing 210-037, China

[2] Department of Biotechnology, Pukyong National University, Busan 48513, Republic of Korea

*** Corresponding authors**

Jong Min Lee

E-mail: jmlee84@pknu.ac.kr

Long Jin

Tel: +86-25-8542-7210

E-mail: isacckim@alumni.kaist.ac.kr

Figure S1. ANI analysis of SN-4-17.

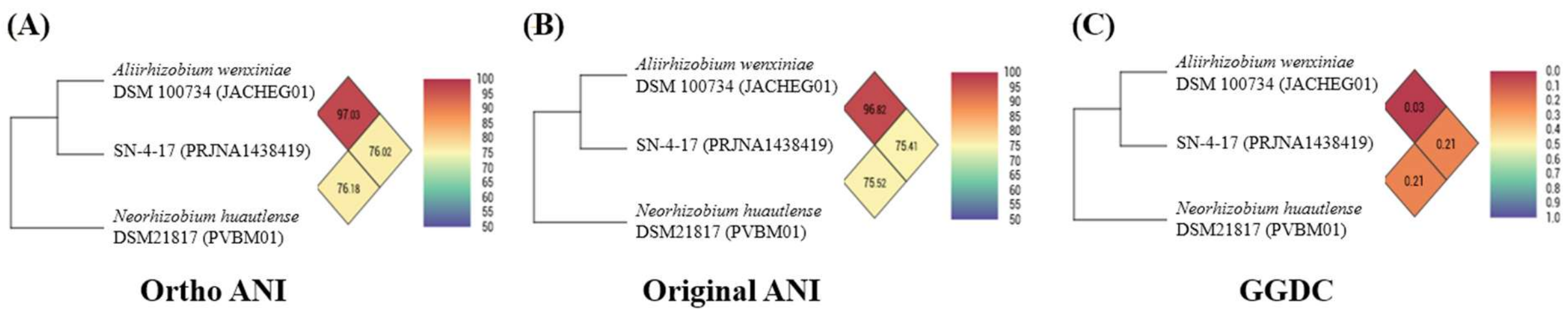

Figure S2. Growth phenotypes of SN-4-17.

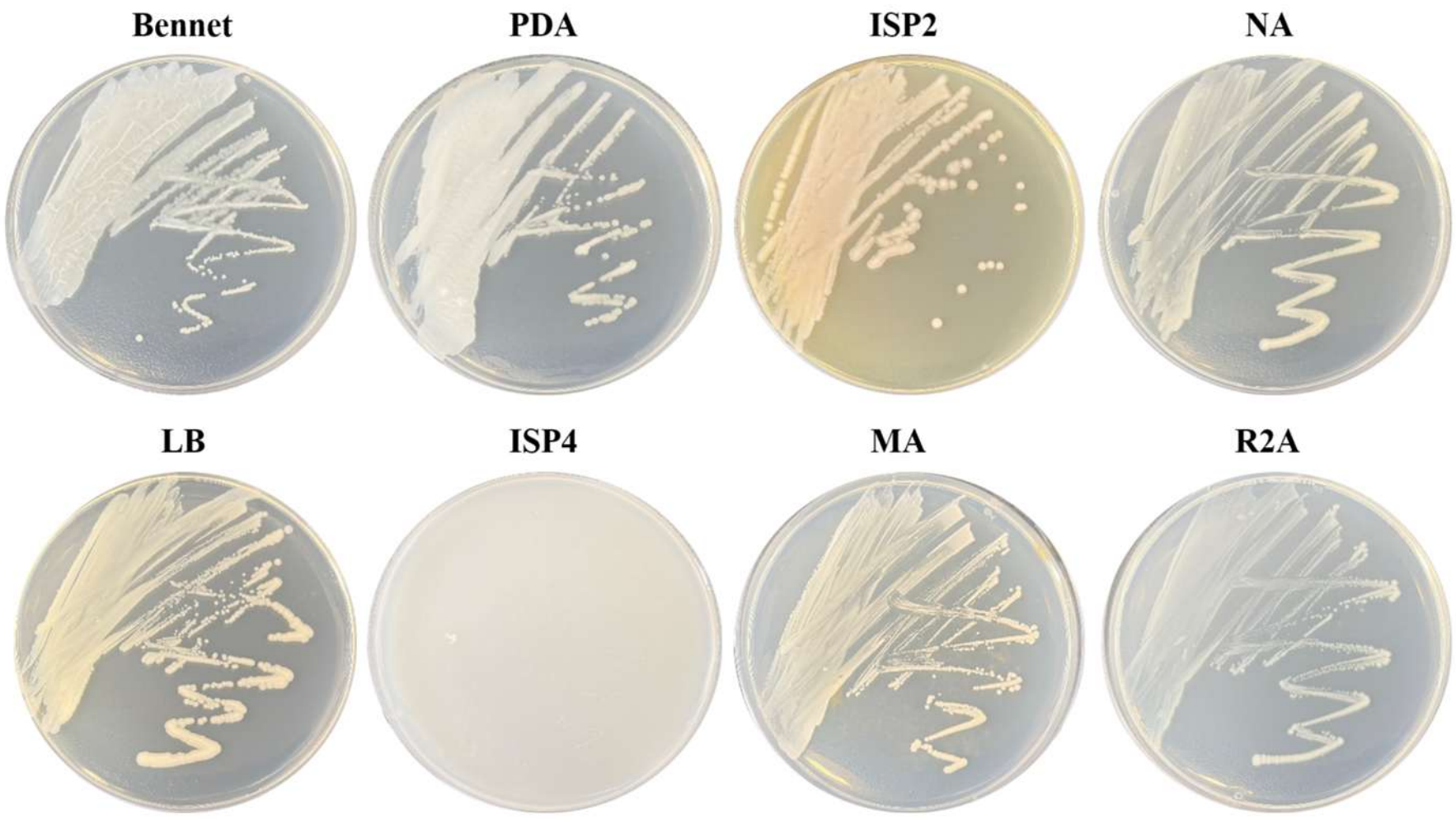

Figure S3. Standard curve of phosphatase.

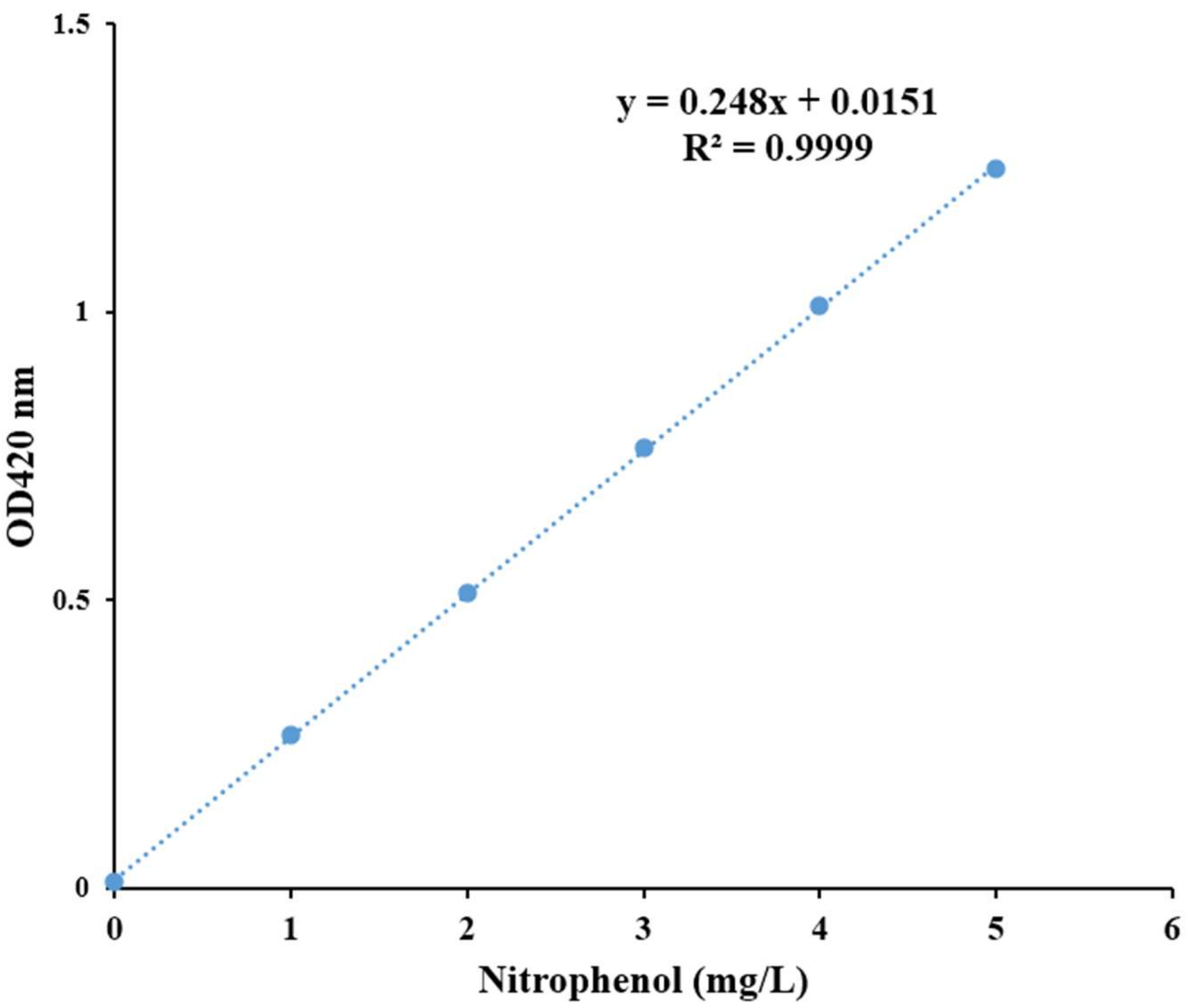

Table S1. Pot experiment design.

Group	Inoculation (mL)	$CaPO_4$ (g/pot)
Water	3	0
Water	3	2
SN-4-17	3	0
SN-4-17	3	2

Table S2. General growth and physiology characteristics of SN-4-17.

Characteristics	SN-4-17
Growth temperature (optimum, °C)	10 – 40 (28)
NaCl tolerance (optimum, %)	0 – 2.0 (0)
Growth pH (optimum)	5.0 – 9.0 (7.0)

Table S3. Characterization of SN-4-17 using API ZYM.

API ZYM	Substrate	Activity
Alkaline phosphatase	2-Naphthyl phosphate	-
Esterase (C4)	2-Naphthyl butyrate	-
Esterase (C8)	2-Naphthyl caprylate	+
Lipase (C14)	L-leucyl-2-naphthylamide	+
Leucine arylamidase	L-leucyl-2-naphthylamide	-
Valine arylamidase	L-Valyl-2-naphthylamide	-
Cystine arylamidase	L- ystyl-2-naphthylamide	+
Trypsin	Benzoyl-DL-arginine-2-naphthylamide	-
α-Chymotrypasin	Glutaryl-phenylanine-2-naphthylamide	+
Acid phosphatase	2-Naphtyl phosphate	-
Naphthol-AS-BI-	Naphthol-AS-BI-phosphate	-
α-Galactosidase	Br-2-naphthyl-*α*-D-galactopyranoside	+
β-Galactosidase	2-NaphthyI-*β*-D-galactopyranoside	+
β-Glucuronidase	Naphthol-AS-BI-*β*-D-glucuronide	+
α-Glucosidase	Naphthyl-α-D-glucopyranoside	-
β-Glucosidase	6-Br-2-naphthyl-*β*-D-glucopyranoside	-
N-Acetyl-*β*-glucosaminidase	1-Naphthyl-N-acetyl-*β*-D-glucosaminide	+
α-Mannosidase	Br-2-naphthyl-*α*-D-mannopyranoside	+
α-Fucosidase	Naphthyl-*α*-L-fucopyranoside	+

Table S4. Characterization of SN-4-17 using API 20 NE.

API 20NE	Function/Substrate	Substrate	Activity
NO2	Reduction of nitrates to nitrites	Potassium nitrate	+
N2	Reduction of nitrates to nitrogen	-	-
TRP	Indole production	Tryptophanes	-
GLU	Acidification	Glucose	-
ADH	Arginine dihydrolase	Arginine	-
URE	Urease	Urea	+
ESC	Hydrolysis (β-glucosidase)	Esculin	+
GEL	Hydrolysis (protease)	Gelatine (with Indiaink)	-
PNPG	β-Galactosidase	*p*-Nitrophenyl-β-D-galactopyranoside	+
GLU	Assimilation	D-Glucose	+
ARA	Assimilation	L-Arabinose	+
MNE	Assimilation	D-Mannose	+
MAN	Assimilation	D-Mannitol	+
NAG	Assimilation	*N*-acetyl-glucosamine	+
MAL	Assimilation	maltose	w
GNT	Assimilation	Gluconate (potassium gluconate)	+
CAP	Assimilation	Caprate (capric acid)	-
ADI	Assimilation	Adipate (adipic acid)	-
MLT	Assimilation	Malate (malic acid)	+
CIT	Assimilation	Citrate (trisodium citrate)	-
PAC	Assimilation	Phenyl acetate (phenylacetic acid)	-

Table S5. Genome information of SN-4-17.

Attribute	Value
Chromosome (bp)	6,409,551
Total gene length (bp)	5,651,465
N50 value	3,973,541
GC content (%)	58.66
Number of genes	6,097
Genes assigned to COG	4,990
Genes with Pfam domains	4,994
tRNA genes	63
rRNA genes (5S/16S/23S)	16 (6/5/5)
Gene island	16
Repeat sequence	75
